# Quantitative Threshold Analysis: Assessing Breastfeeding’s Protective Impact on Bronchopulmonary Dysplasia in Preterm Infants

**DOI:** 10.1155/jonm/5560949

**Published:** 2026-07-12

**Authors:** Mengqing Pan, Yijia Chen, Xiaoyu Lu, Chuntian Liu, Xiaochun Chen, Xiaojing Hu

**Affiliations:** ^1^ School of Nursing, Wenzhou Medical University, Wenzhou, China, wmu.edu.cn; ^2^ Department of Neonatology, The Second Affiliated Hospital and Yuying Children’s Hospital of Wenzhou Medical University, Wenzhou, China, wmu.edu.cn; ^3^ Department of Neonatology, Children’s Hospital of Fudan University, Shanghai, China, fudan.edu.cn

**Keywords:** breastfeeding, bronchopulmonary dysplasia, preterm infants, protective, threshold analysis

## Abstract

**Objective:**

This study aims to thoroughly investigate the protective effects of various breastfeeding rates on bronchopulmonary dysplasia (BPD), clarify the relationship between them, and further determine the minimum protective threshold.

**Methods:**

This prospective study enrolled 276 preterm infants who were born in the obstetrics department of a tertiary Grade A hospital in Zhejiang, China, and were directly admitted to the NICU.

**Results:**

This study involved 276 infants, categorized into three breastfeeding groups based on proportion: low (100 infants, 36.2%), medium (98 infants, 35.5%), and high (78 infants, 28.3%). Notably, the incidence of BPD in the high‐proportion breastfeeding group (19.5%) was significantly lower than that in the medium‐proportion group (44.5%) and the low‐proportion group (55%) (*p* < 0.05); however, there was no statistically significant difference in the incidence of BPD between the medium‐proportion group and the low‐proportion group (*p* > 0.05). Exploratory nonlinear analyses suggested a possible candidate inflection point around 0.31, above which the inverse association between breastfeeding proportion and BPD appeared stronger.

**Conclusion:**

Breastfeeding plays a crucial role in protecting premature infants from BPD. However, this protective effect does not increase linearly but becomes significant only when the breastfeeding rate reaches 31%. However, this threshold‐related finding should be interpreted cautiously and requires validation in larger studies with more comprehensive confounder adjustment.

## 1. Introduction

Preterm birth represents a significant global health challenge, with an incidence of approximately 11% worldwide [1] and 5–10% in China. Despite advancements in perinatal medical technology and neonatal care that have markedly improved the survival rates of preterm infants, these babies, particularly those with extremely low birth weight (ELBW, less than 1000 g), face higher mortality rates and long‐term health issues due to incomplete organ development [2]. Bronchopulmonary dysplasia (BPD) is one of the most common and severe complications, profoundly affecting long‐term pulmonary function, neurodevelopmental outcomes, and overall quality of life [3–5]. The younger the gestational age and the lower the birth weight, the higher the incidence of BPD. Studies indicate that the incidence rate is about 39% for preterm infants born before 32 weeks of gestation, while for extremely low birth weight infants (ELBWIs), it reaches as high as 72.2% [6].

Over the years, research has demonstrated that breastfeeding plays a crucial role in promoting lung development and reducing the risk of BPD [7, 8]. Human milk provides essential nutrients, bioactive factors, and immune components that enhance pulmonary maturation [9], regulate inflammatory responses, and improve long‐term respiratory outcomes. Numerous studies have shown that exclusive breastfeeding can decrease the incidence and severity of BPD, with a dose‐dependent protective effect [10, 11]. Notably, our previous research found that exclusive human milk feeding reduced the incidence of BPD by 10.4% in very low birth weight infants (VLBWIs) [12], and early colostrum administration within 24 h of birth lowered the risk of BPD by 11.25% in mechanically ventilated ELBWIs [11].

When maternal milk is insufficient, donor human milk (DHM) becomes a vital alternative for preterm infants. Multiple studies indicate that using donated breast milk significantly reduces the incidence of necrotizing enterocolitis (NEC) [13] and, to some extent, decreases infections and inflammatory responses, thereby indirectly lowering the risk of BPD [14]. DHM contains multiple immunologically active components and growth factors. Although some components lose activity after pasteurization [15], it remains superior to formula milk and offers significant protective benefits for the lungs of preterm infants. Meanwhile, multiple regions across China are actively establishing breast milk banks. To date, 14 additional banks have commenced operations in major cities across southern, eastern, northern, central, and southeastern China [16]. Relevant research analysis indicates that both the number of breast milk donors and the total volume of donated milk in China show a significant upward trend. Donated breast milk is used not only for premature infants but also for other sick children [17]. Accordingly, in settings of limited MOM, DHM—procured through accredited milk banks and provided with human‐milk–derived or individualized fortification to meet protein–energy targets—should be integrated into standardized NICU feeding pathways and BPD prevention bundles [18].

Even though there is strong proof that breastfeeding helps prevent BPD, the use of breastfeeding in neonatal intensive care units (NICUs) in China is still not good, with less than 15% of hospitalized preterm infants being exclusively breastfed [11]. Existing barriers include NICU policy restrictions, mother–infant separation, and insufficient breastfeeding support [19]. To further optimize breastfeeding strategies for hospitalized preterm infants, this study aims to explore the protective effects of different breastfeeding ratios on BPD and analyze potential quantitative thresholds.

## 2. Methods

### 2.1. Study Design

This study is a prospective investigation. According to the early enteral nutrition status of preterm infants in the NICU and the breastfeeding classification method, the breastfeeding groups were divided into high‐proportion, medium‐proportion, and low‐proportion categories. The total feeding volume was extracted from nursing records, while the breastfeeding volume was obtained from the closed‐loop breastfeeding management system. The proportion of breastfeeding in the total feeding volume was calculated from admission to 28 days after birth. Among them, a breastfeeding proportion of ≥ 80% of the total feeding volume was defined as high‐proportion breastfeeding (including exclusive breastfeeding), 20% to < 80% as medium‐proportion breastfeeding, and < 20% as low‐proportion breastfeeding (including formula feeding). At discharge, we reviewed the breastfeeding records in the department’s closed‐loop breastfeeding management system and documented the breastfeeding status for each infant.

### 2.2. Study Participants

This study enrolled preterm infants admitted to the neonatal ward of a tertiary Grade A hospital in Wenzhou between October 1, 2022, and October 31, 2024. The inclusion criteria include ① gestational age of 28–32 weeks and birth weight of 1000–1500 g, ② birth in the hospital’s obstetrics department followed by direct admission to the neonatal ward, and ③ initiation of early enteral nutrition within 12 or 48 h after birth. Exclusion criteria comprised ① mothers with breastfeeding contraindications (e.g., active pulmonary tuberculosis, HIV, or breast diseases), ② infants born with complications such as congenital heart disease, neonatal birth defects, or intracranial hemorrhage, and ③ discharged cured but readmitted within 1 month. The dropout criteria were ① infants who withdrew from the study midway and ② infants whose families opted to discontinue treatment due to the infant’s condition.

### 2.3. Sample Size Determination

This study is a prospective study that categorizes participants into three groups based on the proportion of breast milk feeding: high‐proportion, moderate‐proportion, and incidence of BPD. Based on the literature review [20, 21], the expected BPD incidence rates are 3% in the high‐proportion group, 15% in the moderate‐proportion group, and 30% in the low‐proportion group. With a two‐sided α of 0.05 and a power of 80%, the calculated total sample size required is 204 cases. Considering a 20% invalid sample rate, the final minimum required sample size is 255 cases, and a total of 276 participants were ultimately enrolled in the study.

### 2.4. General Information Questionnaire

A systematic search was conducted across multiple databases, including CNKI, Wanfang, VIP, and PubMed, to obtain relevant literature and support variable selection. Drawing on the evidence from the literature, combined with clinical experience and expert consultation, key variables were identified and used to develop a comprehensive general information questionnaire.

The questionnaire comprises three main sections. The first section focuses on demographic information, including infant sex, gestational age, birth weight, mode of delivery, and length of hospital stay. The second section addresses clinical characteristics, such as the diagnosis of BPD and major treatments received during hospitalization, including oxygen therapy and mechanical ventilation. The third section captures feeding status, specifically the proportion of breast milk feeding.

### 2.5. Study Processes

#### 2.5.1. Nutritional Protocol

Parenteral nutrition (PN) support was initiated within 24 h after birth, with adjustments based on gestational age, birth weight, postnatal age, and clinical condition. The PN volume was gradually reduced in response to enteral feeding progression and discontinued once enteral intake reached 120 mL/(kg·d) or enteral caloric intake reached 90 kcal/(kg·d). PN solutions were uniformly prepared by the hospital’s centralized intravenous nutrition compounding center using an “all‐in‐one” formulation.

Enteral nutrition: (1) Initiation of Feeding: For VLBW and ELBW preterm infants without intrauterine distress and in stable condition, feeding commenced within 12 h postbirth. For those with intrauterine distress or instability, initiation was delayed between 12 and 24 h, or extended to 24–48 h as appropriate. (2) Initial Feeding Volume: The initial feeding volume for VLBW preterm infants is 1–2 mL every 2 h (q2h), while for ELBW preterm infants, it is 0.5–1 mL every 2 h (q2h). (3) Feeding Advancement Protocol: During the first 1–4 days after birth, minimal enteral feeding should be implemented. If feeding tolerance is achieved, the feeding volume for VLBW preterm infants should be increased at a rate of 20–30 mL/(kg·d), and for ELBW preterm infants, at a rate of 15–25 mL/(kg·d), until the total feeding volume reaches 150–180 mL/(kg·d) or the caloric intake reaches 110–130 kcal/(kg·d) (1 kcal = 4.184 kJ). (4) Choice of Milk Formula: For enteral nutrition, breastfeeding is the first choice, and insufficient breast milk is supplemented with premature infant formula.

#### 2.5.2. Implementation of Breastfeeding

##### 2.5.2.1. Breastfeeding Management

All procedures, including breast milk feeding education, management, and administration, were rigorously adhered to according to the “Evidence‐Based Guidelines for Breastfeeding of Hospitalized Newborns in China.” Our department provides ongoing breastfeeding education and is equipped with a standardized breast milk room, dedicated storage facilities (medical‐grade dual‐temperature refrigerators), and a dedicated breast milk management nutritionist. All breast milk, from collection at home to reception, storage, preparation, and feeding in the hospital, is managed through our hospital’s information‐based closed‐loop management system.

##### 2.5.2.2. Breastfeeding Education

NICU collaborates with obstetric nurses to provide breastfeeding education. During the mother’s stay in the obstetrics department, obstetric nurses, following standard nursing protocols, provided bedside education and guidance on breastfeeding and assisted with the use of breast pumps. Under the professional guidance of the obstetric nurses, parents or dedicated caregivers of premature infants regularly delivered breast milk to our Neonatology Department. On the day of the infant’s admission, the responsible neonatal nurses conducted specialized training on breast milk collection, storage, and transportation (including operational demonstrations and risk assessments) for the parents or caregivers. After the training, an informed consent form for breastfeeding was signed. Following the mother’s discharge from the obstetrics department, family members used cold chain equipment to maintain the cold chain during the transportation of breast milk to our department.

Families are guided to subscribe to our dedicated breastfeeding WeChat account and engage in systematic learning through the platform’s illustrated and video tutorials. This aims to enhance their home breast milk management skills, ultimately better supporting the breastfeeding of their hospitalized preterm infants.

##### 2.5.2.3. Breast Milk Management

After admission, nurses provide education and training to families on breastfeeding knowledge and procedures, and implement a closed‐loop management system to achieve full‐process informatization of breast milk from collection, storage, and portioning to feeding, ensuring real‐time synchronization of information across all stages with the nursing and medical order systems.

Upon admission, the neonatal ward provides a dedicated breastfeeding QR code for the family of each hospitalized infant, and each breast milk storage container is labeled with a unique QR code. During each stage of receiving, storing, portioning, and feeding in the neonatal ward, neonatal nurses use personal digital assistants (PDAs) to scan the QR codes for verification and recording, ensuring transparency and safety throughout the process.

##### 2.5.2.4. Breastfeeding Methods

Follow the doctor’s orders for breastfeeding. When the milk intake reaches 80–100 mL/(kg·d), human milk fortifiers are introduced, initially at half the full dose, with a gradual transition to full fortification over 3–5 days.

In cases of insufficient breast milk supply, preterm infant formula, uniformly procured by the hospital, is used. The dietitian prepares the formula according to the prescribed volume, and the bedside nurse administers the feeding.

### 2.6. Data Collection

Data collection was conducted by two systematically trained master’s students, strictly adhering to the predetermined inclusion and exclusion criteria. To ensure data accuracy and completeness, the researchers performed double‐checking before entering the data into an Excel spreadsheet. Any issues arising during data analysis were promptly consulted with statistical experts to guarantee proper interpretation and processing. All data were securely stored by designated personnel to ensure data security and confidentiality.

### 2.7. Statistical Analysis

All statistical analyses were performed using SPSS software (Version 27.0). Normally distributed continuous variables were presented as mean ± standard deviation (mean ± SD) and compared using one‐way analysis of variance (ANOVA). Non‐normally distributed continuous variables were expressed as median and interquartile range [M (Q1, Q3)] and compared using the Kruskal–Wallis test. Categorical variables were presented as counts and percentages and compared using the chi‐square test. For variables with significant overall group differences, post hoc pairwise comparisons were performed with Bonferroni adjustment.

Univariable logistic regression analyses were first performed for candidate variables associated with BPD. Variables of clinical relevance and/or baseline imbalance were then entered into multivariable logistic regression models, with BPD as the dependent variable and breastfeeding proportion as the primary independent variable. The multivariable model was adjusted for gestational age, birth weight, invasive mechanical ventilation, noninvasive mechanical ventilation, early oral colostrum administration, antenatal corticosteroid exposure, and prenatal antibiotic exposure. Odds ratios (ORs) or adjusted odds ratios (aORs) with 95% confidence intervals (CIs) were reported. Model fit was assessed using the Hosmer–Lemeshow goodness‐of‐fit test.

The association between breastfeeding proportion and BPD was further explored using a generalized additive model (GAM) with a logit link. This nonlinear threshold analysis was exploratory and based on the crude association between breastfeeding proportion and BPD. A piecewise regression model was used to identify a candidate inflection point by likelihood‐based estimation, and bootstrap resampling (1000 iterations) was performed to assess the stability of the segment‐specific estimates above and below the candidate inflection point.

A two‐sided *p* value < 0.05 was considered statistically significant.

### 2.8. Ethical Considerations

This study was approved by the hospital’s Ethics Committee (Approval No.: 2023‐K‐239‐01). The participants in this study included infants and their parents. Throughout the research process, special attention was given to privacy protection and informed consent to ensure that participants’ rights were fully safeguarded.

## 3. Results

### 3.1. Sample Characteristics

This study initially included 290 preterm infants, with 4 deaths and 10 cases with incomplete data. A total of 276 preterm infants were ultimately included: 148 males (53.6%) and 128 females (46.4%); gestational age ranged from 28 to 32 (29.72 ± 2.12) weeks; birth weight ranged from 1000 to 1500 (1173.46 ± 213.31) g; 198 cases (71.7%) were delivered via cesarean section and 78 cases (28.3%) via vaginal delivery; and among them, 100 cases (36.2%) received low‐frequency breastfeeding, 98 cases (35.5%) received moderate‐frequency breastfeeding, 98 cases (35.5%), and high‐proportion breastfeeding in 78 cases (28.3%), as shown in Table 1.

**TABLE 1:** Comparison of baseline characteristics of the study participants.

Factor	Breastfeeding proportion			Test statistic	*p* value
	Low (*n* = 100)	Moderate (*n* = 99)	High (*n* = 77)		
Gender, *n* (%)				5.096^1^	0.078
Female	41 (41)	43 (43.4)	44 (57.1)		
Male	59 (59)	56 (56.6)	33 (42.9)		
Duration of mechanical ventilation (M [Q1, Q3])	26 (10, 36)	30 (12, 45)	21 (8, 35.5)	3.257^2^	0.196
Mode of delivery, *n* (%)				0.947^1^	0.623
Cesarean section	72 (72.0)	68 (68.7)	58 (75.3)		
Vaginal delivery	28 (28.0)	31 (31.3)	19 (24.7)		
Gestational age (M [Q1, Q3])	29.14 (28.57, 30.71)	29.42 (28.42, 31.00)	30.00 (28.93, 30.14)	5.527^2^	0.063
Birth weight (M [Q1, Q3])	1170 (1061.25, 1297.5)	1100 (1040, 1300)	1280 (1120, 1415)	15.170^2^	< 0.001
1‐min Apgar score (M [Q1, Q3])	8 (7, 9)	8 (7, 9)	8 (7.5, 9)	2.501^2^	0.286
5‐min Apgar score (M [Q1, Q3])	9 (9, 9)	9 (9, 9)	9 (9, 10)	2.453^2^	0.293
Early oral colostrum administration [*n* (%)]				46.806^1^	< 0.001
No	86 (86.0)	64 (64.6)	28 (36.4)		
Yes	14 (14.0)	35 (35.4)	49 (63.6)		
Prenatal use of corticosteroids [*n* (%)]				8.831^1^	0.012
No	19 (19.0)	35 (35.3)	15 (19.5)		
Yes	81 (81.0)	64 (64.7)	62 (80.5)		
Prenatal use of antibiotics [*n* (%)]				7.262^1^	0.026
No	59 (59.0)	53 (53.5)	30 (38.9)		
Yes	41 (41.0)	46 (46.5)	47 (61.1)		

^1^Chi‐squared value.

^2^F value.

### 3.2. Comparison of Baseline Characteristics Among Different Breastfeeding Proportion Groups

There were no statistically significant differences (*p* > 0.05) among the three groups of preterm infants in terms of gender, gestational age, duration of mechanical ventilation, mode of delivery, 1‐min Apgar score, and 5‐min Apgar score. There were statistically significant differences in birth weight, BPD, colostrum oral smear, prenatal corticosteroid use, and prenatal antibiotic use (*p* < 0.05), as shown in Table 1.

### 3.3. Comparison of BPD Incidence Across Different Breastfeeding Proportion Group

**FIGURE 1:**
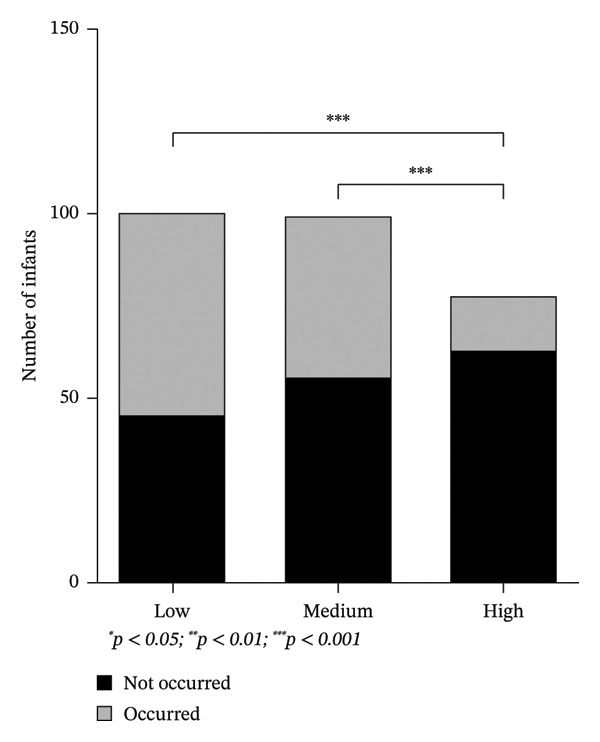
The relationship between breastfeeding and the incidence of bronchopulmonary dysplasia in different proportions.

The incidence of BPD in the high‐proportion breastfeeding group (19.5%) was significantly lower than that in the medium‐proportion group (44.5%) and the low‐proportion group (55%) (*p* < 0.05); however, there was no statistically significant difference in the incidence of BPD between the medium‐proportion group and the low‐proportion group (*p* > 0.05). Detailed results are shown in Figure 1.

### 3.4. Analysis of the Relationship Between Breastfeeding Proportion and BPD

#### 3.4.1. Multivariable Logistic Regression Analysis of Factors Associated With BPD

Univariable logistic regression analysis showed that human milk proportion, gestational age, birth weight, colostrum oropharyngeal administration, and invasive mechanical ventilation were significantly associated with the occurrence of BPD. Multivariable logistic regression analysis further demonstrated that after adjusting for gestational age, birth weight, antenatal corticosteroid use, antenatal antibiotic use, colostrum oropharyngeal administration, invasive mechanical ventilation, and noninvasive mechanical ventilation, human milk proportion remained significantly associated with the occurrence of BPD (aOR = 0.262, 95% CI: 0.103–0.667, *p* = 0.005). In addition, gestational age and birth weight were significantly associated with lower odds of BPD, whereas invasive mechanical ventilation was significantly associated with higher odds of BPD. Detailed results are shown in Table 2.

**TABLE 2:** Univariable and multivariable logistic regression analyses of factors associated with BPD.

Factor	Single factor OR (95% CI)	*p* value	Multifactorial OR (95% CI)	*p* value
Proportion of breast milk	0.185 (0.094–0.364)	< 0.001	0.262 (0.103–0.667)	0.005
Gestational age	0.427 (0.336–0.543)	< 0.001	0.488 (0.367–0.650)	< 0.001
Birth weight	0.995 (0.993–0.997)	< 0.001	0.997 (0.995–0.999)	0.006
Prenatal use of corticosteroids	0.961 (0.553–1.670)	0.888	0.940 (0.459–1.927)	0.866
Prenatal use of antibiotics	1.040 (0.644–1.679)	0.873	0.977 (0.514–1.856)	0.943
Early oral colostrum administration	0.460 (0.272–0.777)	0.004	1.256 (0.626–2.522)	0.521
Invasive mechanical ventilation	1.110 (1.069–1.152)	< 0.001	1.097 (1.053–1.144)	< 0.001
Noninvasive mechanical ventilation	1.009 (0.995–1.024)	0.212	1.012 (0.993–1.032)	0.203

*Note:* The multivariable logistic regression model included breastfeeding proportion, gestational age, birth weight, antenatal corticosteroid exposure, prenatal antibiotic exposure, colostrum oral care, invasive mechanical ventilation, and noninvasive mechanical ventilation. The Hosmer–Lemeshow test indicated acceptable model fit (χ^2^ = 6.996, *p* = 0.537).

Abbreviations: aOR, adjusted odds ratio; CI, confidence interval; OR, odds ratio.

#### 3.4.2. Smooth Curve Fitting Analysis

**FIGURE 2:**
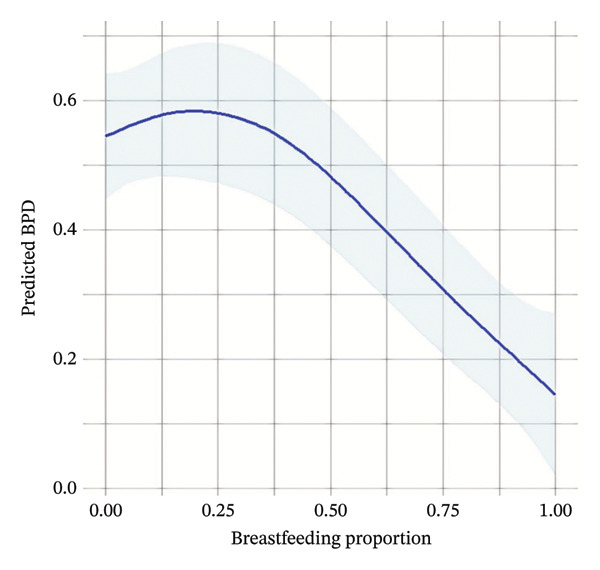
Smooth curve fitting for the association between breastfeeding proportion and bronchopulmonary dysplasia. A generalized additive model (GAM) was used to explore the association between breastfeeding proportion and bronchopulmonary dysplasia (BPD). The solid blue line represents the fitted curve, and the shaded area indicates the 95% confidence interval. The curve suggests a potential nonlinear association between breastfeeding proportion and BPD.

A GAM was used to perform a smooth curve fit of the relationship between the proportion of breast milk and BPD. The results indicate that there may be a nonlinear association between the proportion of breast milk and BPD (Figure 2). The curve suggests that the probability of BPD changes little within the low‐to‐moderate ranges of breast milk intake, whereas it shows a downward trend in the higher ranges. Based on these findings, a piecewise regression model was further employed to identify potential inflection points.

#### 3.4.3. Candidate Inflection Point Estimation

Based on the model fitting results, the maximum likelihood method was used to estimate the candidate inflection point in the relationship between the breastfeeding rate and BPD. The results suggest that this candidate inflection point is approximately 0.31 (Table 3). Given that this analysis is an exploratory nonlinear analysis, this value should be interpreted as a candidate inflection point rather than a definitive clinical threshold.

**TABLE 3:** Candidate inflection point and segmented regression results for the association between breastfeeding proportion and bronchopulmonary dysplasia.

Change point (psi)	Estimate (Est.)	95% confidence interval (95% CI)
0.31	0.04	[0.4262, 0.6393]

*Note:* ψ, candidate inflection point. The candidate inflection point was estimated using likelihood‐based methods. Segment‐specific regression coefficients are presented for observations below and above ψ. Given the exploratory nature of the nonlinear analysis, ψ = 0.31 should be interpreted as a candidate inflection point rather than a definitive clinical threshold.

#### 3.4.4. Segmented Regression Analysis

**FIGURE 3:**
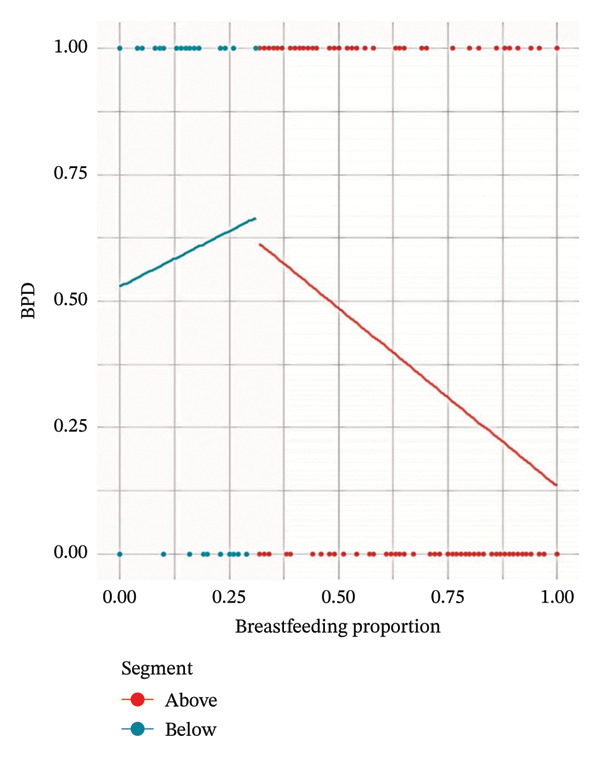
Segmented regression analysis of the association between breastfeeding proportion and bronchopulmonary dysplasia. Segmented regression analysis was performed using the candidate inflection point ψ = 0.31. Different colored lines represent the fitted trends below and above the candidate inflection point. The association between breastfeeding proportion and BPD did not reach statistical significance below ψ, whereas a negative association was observed above ψ. These findings support the possibility of a nonlinear relationship between breastfeeding proportion and BPD.

Segmented regression analysis was performed using 0.31 as the candidate inflection point. The results showed that within the range where the breastfeeding proportion was below 0.31, the association between the breastfeeding proportion and BPD was not statistically significant; however, within the range where the breastfeeding proportion was above 0.31, the breastfeeding proportion was significantly negatively correlated with BPD (Figure 3). These findings suggest that the negative correlation between the breastfeeding proportion and BPD may be more pronounced at higher levels of breastfeeding.

#### 3.4.5. Model Comparison

Further comparison of the fit between segmented regression models incorporating candidate inflection points and simple linear models suggests that the former fits better than the latter, supporting the possibility of a nonlinear relationship between the proportion of breast milk and BPD. However, given that this study is observational and residual confounding factors may still be present, these results should be considered exploratory findings.

#### 3.4.6. Bootstrap Resampling Evaluation

**FIGURE 4:**
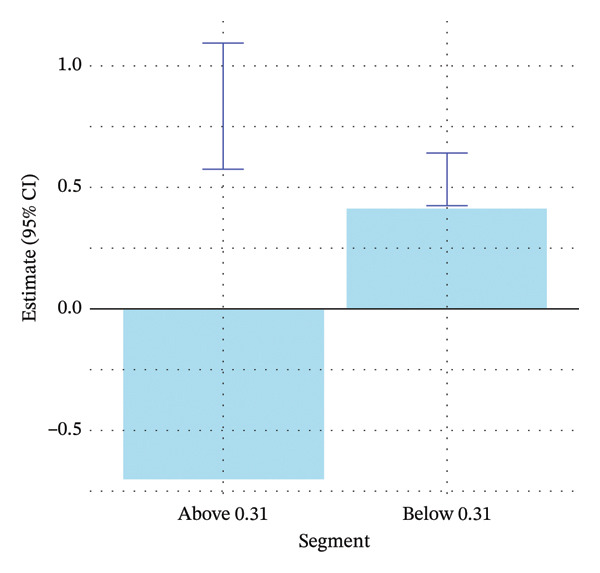
Bootstrap resampling assessment of the segmented regression results. Bootstrap resampling (1000 iterations) was used to assess the robustness of the segmented regression results. The figure presents the segment‐specific regression coefficients and their 95% confidence intervals below and above the candidate inflection point ψ = 0.31. The direction of the coefficient above ψ appeared relatively stable, whereas the estimate below ψ was less stable. These results were used to assess the stability of the exploratory nonlinear analysis and should not be interpreted alone as evidence of a definitive clinical cutoff.

We conducted a robustness analysis of the segmented regression results using 1000 bootstrap resamples. The results showed that the direction of the segmented regression coefficients was relatively stable for the segment above the candidate inflection point, whereas the estimates for the segment below the candidate inflection point were unstable and did not reach statistical significance. These findings further support the notion that the negative correlation between breast milk proportion and BPD may be more consistent within the higher breast milk proportion range; however, this alone is insufficient to serve as the basis for determining a clinical threshold. Detailed results are shown in Figure 4.

Overall, the association between breastfeeding proportion and BPD may be nonlinear, with an exploratory candidate inflection point around 0.31. Above this value, the inverse association between breastfeeding proportion and BPD appeared stronger. Nevertheless, this finding requires further validation in larger studies with more comprehensive adjustment for potential confounders.

#### 3.4.7. Subgroup Analysis by Gestational Age

In this study, no significant differences in gestational age were observed when comparing groups with different rates of breastfeeding; however, as a key baseline characteristic of preterm infants, gestational age is widely recognized as being closely associated with the risk of BPD. Given that newborns at different gestational ages differ in terms of lung development, immune function, and feeding tolerance, these factors may influence the protective effect of breastfeeding against BPD. Therefore, to further explore the consistency and potential heterogeneity of breast milk’s protective effect against BPD across different gestational age subgroups, this study proposes to conduct a subgroup analysis stratified by gestational age to assess its moderating role.

**FIGURE 5:**
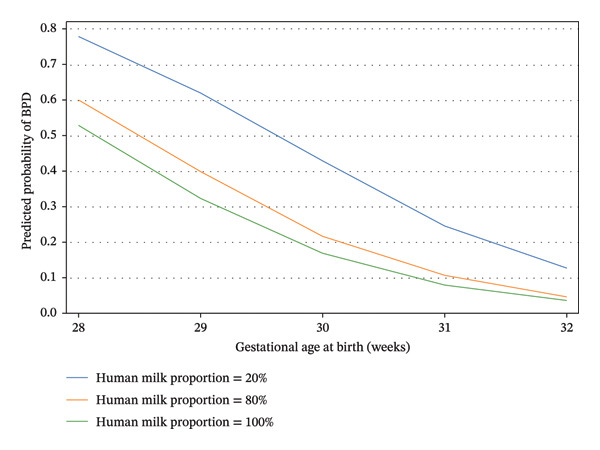
Prevalence of BPD: subgroup analysis of breastfeeding rates and gestational age.

Although the interaction term between breast milk proportion and gestational age did not reach statistical significance (*p* = 0.985), suggesting that the absolute difference in the protective effect of breast milk across different gestational age subgroups may not exceed the range of random variation, marginal effects analysis revealed a trend with potential clinical significance: among 28‐week preterm infants, a change in breast milk proportion from 20% to 100% the relative risk of BPD decreased by 27.4%. Among 30‐week preterm infants, the same change resulted in a 26.2% reduction in the relative risk of BPD. Among 32‐week preterm infants, the same change resulted in a 13.8% reduction in the relative risk of BPD. Detailed results are shown in Figure 5.

This subgroup analysis suggests that although the protective effect of breastfeeding exhibits a threshold (0.31) at the overall level and the direction of this effect is consistent across preterm infants of different gestational ages (all showing a protective effect), the magnitude of this protection appears to be most pronounced among the youngest preterm infants. This supports the potential additional benefits of implementing breastfeeding strategies with a higher proportion of breast milk for extremely preterm infants. Although this finding requires further research to validate, it provides guidance for developing individualized feeding strategies in clinical practice.

## 4. Discussion

### 4.1. Higher Breastfeeding Proportion Was Associated With Lower Odds of BPD

This study investigated the relationship between varying proportions of breastfeeding and the incidence of BPD in preterm infants, revealing a significant trend wherein higher breastfeeding proportions were associated with lower BPD incidence. Specifically, the incidence rates of BPD were 55%, 44.5%, and 19.5% in the low‐proportion (< 20%), medium‐proportion (20%–80%), and high‐proportion (≥ 80%) breastfeeding groups, respectively. The high‐proportion breastfeeding group showed a significantly lower incidence of BPD (*p* < 0.05), suggesting that a higher breastfeeding proportion was associated with lower odds of BPD.

Extensive research has clearly demonstrated that breastfeeding confers significant protection against BPD in preterm infants. As the optimal source of nutrition for preterm infants [14], breast milk is rich in bioactive components such as immunoglobulins, lactoferrin, and transforming growth factor‐beta (TGF‐β), which promote lung development [22], modulate immune responses, mitigate lung injury caused by mechanical ventilation and oxygen therapy [5, 23], and help alleviate chronic inflammation and fibrosis, thereby reducing the risk of BPD [24]. Nevertheless, many current studies focus solely on whether breastfeeding can prevent the occurrence of BPD, without considering the crucial role of the actual breast milk intake volume.

This study not only suggests an overall association between breastfeeding and lower BPD incidence but also provides a stratified analysis across different breastfeeding proportions. Specifically, this study found that the BPD incidence in the high‐proportion group (breastfeeding proportion > 80%) was significantly lower than in the medium‐ and low‐proportion groups (44.5% and 55%), a finding that is also supported by other studies on preterm infants [25].

#### 4.1.1. Nonlinear Relationship Between Breastfeeding Proportion and BPD

To further explore the relationship between breastfeeding proportion and the incidence of BPD, this study utilized a GAM for smooth curve fitting, revealing a nonlinear relationship between the two. The results showed that when the breastfeeding proportion was below 0.31, the protective effect was not significant (β = 0.4382, *p* = 0.383), but when the proportion exceeded 0.31, the incidence of BPD significantly decreased (β = −0.7041, *p* < 0.001). This finding suggests that the inverse association between breastfeeding proportion and BPD may become more apparent once breastfeeding proportion exceeds a candidate inflection point.

Although the incidence of BPD was lower in the 20%–80% breastfeeding group compared to the low‐proportion group, there was no overall statistically significant difference between the two groups. This may be because the breastfeeding proportions in both groups did not reach a sufficient threshold to trigger the protective effects of breast milk. This also suggests that the benefits of breastfeeding are a gradual process, and its protective effects only become apparent when the feeding proportion approaches or exceeds a certain level (such as 0.31 or higher).

### 4.2. A Candidate Inflection Point in the Association Between Breastfeeding Proportion and BPD

Su et al. [26] reported that breastfeeding proportion may show a threshold‐related pattern in its association with health outcomes, with nonlinear models potentially providing a better fit to the observed data. In the present study, bootstrap resampling (1000 iterations) was used to assess the stability of the segment‐specific estimates, suggesting that the inverse association between breastfeeding proportion and BPD was less stable below approximately 0.31 but appeared more consistent above this value. These findings support the possibility of a dose–response pattern between breastfeeding proportion and BPD incidence, suggesting that the inverse association may become more apparent once a candidate inflection point is surpassed. In particular, BPD incidence was markedly reduced among preterm infants who received breast milk at proportions exceeding 0.31.

The underlying mechanism for this observed association may relate to the role of breast milk in supporting pulmonary development and reducing the need for respiratory support [22, 27]. Preterm infants, due to their immature lungs, often require mechanical ventilation and oxygen therapy to maintain adequate respiration. However, prolonged exposure to these interventions is associated with oxygen toxicity and ventilator‐induced lung injury, both of which increase the risk of BPD [28]. High proportions of breastfeeding not only supply critical nutrients necessary for lung maturation but also support the infant’s ability to achieve autonomous respiration [29], thereby reducing reliance on invasive respiratory therapies and indirectly lowering the risk of BPD [30].

In conclusion, this study reveals the nonlinear relationship between breastfeeding proportion and BPD, showing that high breastfeeding proportions offer protection through multiple mechanisms, including promoting lung development, reducing inflammation, enhancing independent breathing, and regulating the gut–lung axis. Particularly when the breastfeeding proportion exceeds approximately 0.31, the inverse association between breastfeeding proportion and BPD appears stronger, whereas below this candidate inflection point, the association is less evident. Although DHM was not included in this analysis, other studies indicate that when maternal own milk (MOM) is insufficient to reach the minimal protective threshold, supplementing with DHM to achieve that level may be clinically meaningful, sustaining adequate human milk exposure during the early critical window and thereby reducing BPD‐related risk directly or indirectly by lowering NEC/infections [31]. Nevertheless, evidence for a direct DHM effect on BPD remains limited, underscoring the need for high‐quality randomized trials. Collectively, these findings may help inform future studies on feeding strategies and provide a framework for further investigation into the dose–response relationship and possible threshold‐related patterns linking breastfeeding with neonatal respiratory outcomes. A strength of the present study is that breastfeeding proportion was defined within a prespecified early‐life exposure window, from NICU admission to postnatal day 28, which improved temporal ordering between exposure assessment and outcome evaluation and reduced, although did not completely eliminate, concerns about reverse causation.

## 5. Conclusion

A higher breastfeeding proportion was associated with lower odds of BPD in preterm infants; however, this association did not appear to be strictly linear, and exploratory analyses suggested a possible candidate inflection point around 31%. Once this threshold is exceeded, the protective effect of breastfeeding enhances as the rate increases. In light of this finding, for preterm infants who are unable to receive exclusive breastfeeding for various reasons, efforts to optimize breastfeeding management may be considered; however, the value around 31% should not yet be interpreted as a definitive clinical target and requires further validation.

## 6. Limitations of This Study

As a single‐center study, this research has limited external applicability, necessitating further validation of the results through multicenter studies. Furthermore, given the absence of subgroup analysis, there may be variations in the response to breastfeeding among different subgroups of premature infants. Future studies will adopt multicenter designs with prespecified subgroup analyses and will explicitly distinguish MOM from DHM to validate and extend these conclusions.

In addition, the assumed BPD incidence rates used for sample size estimation differed from the observed rates in the final cohort. This discrepancy suggests that the original event rate assumptions were not fully representative of our study population, likely because of differences in case mix and baseline severity, and should be considered when interpreting the initial power calculation.

## Funding

This work was supported by a grant from the Wenzhou Municipal Science and Technology Bureau Project in Zhejiang Province, China (Item No. Y2023049).

## Conflicts of Interest

The authors declare no conflicts of interest.

## Data Availability

Research data are not shared.
