# Correction to “Quantitative Threshold Analysis: Assessing Breastfeeding’s Protective Impact on Bronchopulmonary Dysplasia in Preterm Infants”

**DOI:** 10.1155/jonm/9851543

**Published:** 2026-09-17

M. Pan, Y. Chen, X. Lu, C. Liu, X. Chen, and X. Hu, “Quantitative Threshold Analysis: Assessing Breastfeeding’s Protective Impact on Bronchopulmonary Dysplasia in Preterm Infants,” *Journal of Nursing Management* 2026 (2026): 5560949, https://doi.org/10.1155/jonm/5560949.

In the published article, Affiliation 1 and Affiliation 2 were listed in the incorrect order, resulting in incorrect superscript affiliation numbers for several authors, including the corresponding author. The correct author affiliations and superscript affiliation numbers should read as follows:

Mengqing Pan^1,2^ & Yijia Chen^1,2^, Xiaoyu Lu^2^, Chuntian Liu^2^, Xiaojing Hu^3^, Xiaochun Chen^1∗^,

^1^Department of Neonatology, The Second Affiliated Hospital and Yuying Children’s Hospital of Wenzhou Medical University, Wenzhou, China

^2^School of Nursing, Wenzhou Medical University, Wenzhou, China

^3^Department of Neonatology, Children’s Hospital of Fudan University, Shanghai, China

^∗^Corresponding author: Xiaochun Chen, Department of Neonatology, The Second Affiliated Hospital and Yuying Children’s Hospital of Wenzhou Medical University, No. 1111, Wenzhou Avenue (East), Yaoxi Street, Longwan District, Wenzhou City, Zhejiang Province, China (cherry_mine@126.com)

ORCID iD: https://orcid.org/0000-0003-3385-7230

Mengqing Pan and Yijia Chen contributed equally to this work and should be considered co‐first authors.

We apologize for this error.

